# The Biological Changes of Synaptic Plasticity in the Pathological Process of Sepsis-associated Encephalopathy

**DOI:** 10.2174/1570159X23666241028105746

**Published:** 2024-10-28

**Authors:** Lin Yang, Jin Li, Fuhong Liu, Xin Chai, Zongping Fang, Xijing Zhang

**Affiliations:** 1 Department of Critical Care Medicine, Xijing Hospital, Fourth Military Medical University, Xi’an, Shaanxi, 710032, China;; 2 Department of Critical Care Medicine, Air Force Medical Center, Beijing, 100142, China;; 3 The Third Department of Critical Care Medicine, Shengli Clinical Medical College of Fujian Medical University, Fujian Provincial Hospital, Fuzhou, China;; 4 Translational Research Institute of Brain and Brain-Like Intelligence, Shanghai Fourth People’s Hospital, School of Medicine, Tongji University, Shanghai, China

**Keywords:** Sepsis, sepsis-associated encephalopathy, synaptic plasticity, neuroinflammation, microglia, inflammatory factors

## Abstract

Sepsis-associated encephalopathy (SAE) is a form of cognitive and psychological impairment resulting from sepsis, which occurs without any central nervous system infection or structural brain injury. Patients may experience long-term cognitive deficits and psychiatric disorders even after discharge. However, the underlying mechanism remains unclear. As cognitive function and mental disease are closely related to synaptic plasticity, it is presumed that alterations in synaptic plasticity play an essential role in the pathological process of SAE. Here, we present a systematic description of the pathogenesis of SAE, which is primarily driven by glial cell activation and subsequent release of inflammatory mediators. Additionally, we elucidate the alterations in synaptic plasticity that occur during SAE and comprehensively discuss the roles played by glial cells and inflammatory factors in this process. In this review, we mainly discuss the synaptic plasticity of SAE, and the main aim is to show the consequences of SAE on inflammatory factors and how they affect synaptic plasticity. This review may enhance our understanding of the mechanism underlying cognitive dysfunction and provide valuable insights into identifying appropriate therapeutic targets for SAE.

## 1 INTRODUCTION

Sepsis is a life-threatening syndrome in intensive care units, characterized by fatal dysfunction of organs resulting from an unregulated host reaction to infections [1]. When dysregulation of inflammation involves the nervous system, it causes diffuse brain dysfunction without any infection in the central nervous system (CNS) or structural brain injury, which is known as sepsis-associated encephalopathy (SAE) [2, 3]. Clinically, SAE is characterized by changes in consciousness and mental state, including agitation, hallucinations, decreased attention, delirium, or coma [2]. Patients who have survived SAE warrant special attention because some patients still present with long-term cognitive dysfunction, even after being discharged from the hospital [4, 5]. The remaining neurological problems include alterations in memory, attention, concentration, and even global cognitive function, which seriously jeopardize the quality of life, as well as increase the burden of society [4, 5]. Several large population-based epidemiologic studies, both prospective and retrospective, have shown that sepsis can lead to a substantially increased risk of dementia [6, 7]. However, the mechanism of long-term cognitive impairment and psychiatric changes in septic patients is still unclear. Besides, there are no satisfactory remedies for SAE. Therefore, it is urgent to find some effective treatments for SAE patients.

Several investigations have revealed that cognitive impairment is directly connected to neurodegeneration owing to apoptosis. Meanwhile, evidence of synaptic alterations has also been documented in clinical studies and animal models related to SAE. Some studies have proven that in animals infected with G-bacteria, cognitive function is decreased, but no neuronal death is found. On the other hand, synaptic protein concentration, alpha-amino-3-hydroxy-5-methyl-4 isoxazole-propionic acid receptor (AMPAR), and N-Methyl-D-aspartic acid receptor (NMDAR) expression are reduced, which are closely related with long-term potentiation (LTP) and long-term depression (LTD) [8]. However, the underlying mechanism of the biological process of synaptic plasticity in the pathological process of SAE is not well illustrated.

It is widely accepted that synaptic plasticity refers to the adjustability of information transmission between neurons at the synaptic level, including changes in the synaptic number, structure and transmission efficiency [9]. It has long been recognized that synaptic plasticity is closely related to cognitive function, especially learning and memory [10, 11]. Synaptic plasticity impairment may also be linked to long-term cognitive decline. In this review, we summarize the potential mechanisms of SAE, especially synaptic plasticity changes during sepsis, which would provide new avenues for the development of novel therapeutics for SAE.

## 2 THE MECHANISMS OF SAE

The etiology of SAE is complex and multifaceted, leading to an incomplete understanding of its pathophysiology and underlying molecular mechanisms. Current understanding suggests that pathogenesis involves neuroinflammation, altered cerebral perfusion and microcirculatory disorders, impaired blood-brain barrier (BBB) function, oxidative stress damage, and neurotransmitter alterations [12-14]. Above all, the activation of immune cells within the brain and subsequent release of significant quantities of pro-inflammatory cytokines are believed to constitute the core pathology underlying SAE [15].

Neuroinflammation plays a crucial role in SAE, which is mainly driven by the rapid recruitment of peripheral immune cells and the activation of resident immunologically active cells. Following the onset of sepsis, the brain is affected by inflammatory signals from the periphery *via* both the humoral and neurological systems. Inflammatory mediators can reach the brain directly in BBB-deficient periventricular organs. On the other hand, the vagus nerve conveys local inflammatory signals to the medullary autonomic nuclei [16]. Additionally, some cytokines, including interleukin-1 (IL-1), tumor necrosis factor-α (TNF-α), and interleukin-6(IL-6), can reach the brain through certain cytokine transport receptors [17]. These circulating pro-inflammatory chemicals, especially IL-1, TNF-α and IL-6, promote the upregulation of endothelial cell adhesion molecules [18], which encourages more inflammatory mediators and cells to enter the brain tissue through the damaged BBB. Thereby, inflammatory mediators lead to changes in cells in the brain, such as the activation of microglia and astrocytes [19, 20]. Ultimately, brain dysfunction is exacerbated by the production of pro-inflammatory cytokines from activated glia, which disturb the homeostasis of brain tissue.

Significant cerebral perfusion impairment and microcirculatory abnormalities are also common in SAE patients. Reduced cerebral perfusion during sepsis may be linked to hypotension and disruption of cerebrovascular autoregulation [21]. As already mentioned, reactive oxygen species (ROS) damages endothelial cells, while cytokine storm imbalance between coagulation and anticoagulation induces the production of microthrombi in the cerebral vasculature, resulting in microcirculatory abnormalities [22]. More importantly, neuroinflammation and BBB disruption are mutually causative. On one hand, pro-inflammatory cytokines can act directly on the BBB leading to an increase of permeability. On the other hand, activated astrocytes and microglia can also exacerbate the disruption of the BBB through other pathways. Several data suggest that IL-1β can trigger astrocytes to release vascular endothelial growth factor-A, leading to downregulation of the expression of tight junction proteins between cells [23]. Further, activated microglia can migrate to blood vessels and engulf tight junctions between endothelium.

Oxidative stress is also thought to be an important cause of neuronal loss and dysfunction [24]. Systemic inflammation leads to elevated levels of ROS and RNS, which can inhibit the mitochondrial electron transport chain and destroy the mitochondrial membrane, causing intracellular ATP depletion and neuron dysfunction [25-27]. Besides, cytochrome C released from mitochondria triggers apoptosis, which exacerbates the local inflammatory response further [26]. The aforementioned phenomena manifested following sepsis, with the cortex, hippocampus and cerebellum exhibiting oxidative damage 6 hours post modeling [28]. Targeting this mechanism, antioxidant therapy for sepsis patients has also been explored over the years. Nuclear factor erythroid 2-related factor 2 (Nrf2) is one of the important defense mechanisms in cells against oxidative stress. Studies have shown that hydrogen gas can protect wild-type mice from SAE but not Nrf2 knockout mice [29, 30]. This indicates that Nrf2 is a promising therapeutic target. Besides, there are studies primarily focused on vitamin C due to its prominent role in the anti-oxidative stress function [31-33]. The administration of vitamin C in a rat model of sepsis resulted in enhanced cognitive function and increased survival rates [34]. However, the clinical trial results demonstrated that the combination of vitamin C and thiamine failed to significantly reduce the duration of delirium in septic patients [35], thereby highlighting the complexity of SAE treatment.

Besides the widely accepted mechanisms discussed above, there are many other mechanisms involved in the pathogenesis of SAE, including neurotransmitters and signaling abnormalities. One particularly intriguing mechanism is the dysregulation of cholinergic pathways [36]. Reduced cholinergic activity in sepsis may affect the ability to regulate memory, learning, arousal levels and major cognitive functions [36]. However, cholinesterase inhibitors have not shown efficacy when used for prevention or treatment [22], which indicates that more research is needed to further explore the underlying mechanism.

## 3 SYNAPTIC CHANGES IN SAE

The forms of synaptic plasticity are diverse and can be further categorized into two main types: structural plasticity and functional plasticity [9]. Structural plasticity primarily involves alterations in the quantity and morphology of synapses. The changes can be directly observed in the synaptic structure through an electron microscope or detected through the expression and distribution analysis of synapse-associated proteins, including Synapsin (SYN), Synaptophysin (SYT), and postsynaptic density protein 95 (PSD95). Functional plasticity refers to alterations in synaptic transmission efficiency. Repetitive neuronal stimulation can either enhance or weaken the efficiency of synaptic transmission, leading to the occurrence of LTP and LTD, which are two prominent manifestations of functional plasticity. The alteration of synaptic receptor number and sensitivity is responsible for these phenomena. NMDAR and AMPAR, the two pivotal glutamatergic receptors, play a crucial role in mediating this process [37]. In the case of LTP, repeated high-frequency stimulation induces a substantial release of glutamate, leading to the activation of AMPARs on the postsynaptic membrane and subsequent depolarization. This depolarization facilitates the removal of Mg^2+^ blockage on NMDARs, resulting in an influx of Ca^2+^ into the cell, which serves as a pivotal signal triggering LTP [38]. Intracellular calcium elevation subsequently initiates a cascade of downstream signaling pathways, including CaMKII and PKC, which enhance both the quantity and functionality of AMPARs [38, 39]. These alterations ultimately lead to an amplified postsynaptic neuron response to identical glutamate stimulation, manifesting as LTP. The efficiency of synaptic transmission can be assessed through electrophysiological methods, which directly measure the current or potential changes in postsynaptic neurons.

Evidence of synaptic changes has been observed in both clinical studies and animal models related to SAE. We have compiled a table summarizing the synaptic changes previously documented in animal models of SAE (Table **1**). Previous researches have demonstrated that patients with cognitive impairment exhibit a reduction in hippocampal synaptic density [40-42], indicating that the cognitive deficits observed in SAE patients may be attributed to a decrease in synapse number. In animal models of sepsis, changes in synaptic protein abundance and dendritic spine elimination are observed as a delayed response to LPS injection, accompanied by corresponding alterations in behavior within relevant brain regions [43]. Synaptic loss has been documented in various regions of the brain. After 24 hours of lipopolysaccharide (LPS) injection, reduced expression levels of PSD95 were observed in the hippocampus, prefrontal cortex, and striatum [44].

Moreover, *in vivo* two-photon microscopy showed a reduction of the total spine density in the somatosensory cortex 8 weeks after LPS injection [45]. The hippocampal region has attracted significant attention due to its vulnerability and intimate connection with cognitive function. The type of cognitive impairment is associated with the specific brain region where synaptic loss takes place. For instance, CA1 and the dentate gyrus are respectively linked to long-term spatial memory damage and contextual fear conditioning [46]. However, it should be noted that certain animal experiments have not demonstrated a reduction in dendritic spine density [47], potentially attributable to alterations during the cecal ligation and puncture (CLP) surgical procedure and LPS administration. We should also be aware that synapse loss is a dynamic process, and the changes in different brain regions and the sequence of presynaptic and postsynaptic changes are worthy of further investigation. Current evidence suggests that synaptic loss occurs earlier in hippocampus than in cortex [48], and presynaptic changes precede postsynaptic changes [49].

From the perspective of synaptic functional plasticity, alterations in neuronal spontaneous excitability, LTP and LTD have been reported to be influenced by SAE. The results from the study conducted by Jiang *et al.* showed that spontaneous activity was significantly reduced within the hippocampal CA1 region of CLP mice [50]. The same results were obtained for the peritoneal contamination and infection (PCI) model, indicating a decrease in the frequency of spontaneous mEPSCs [51]. The investigation into the impact of SAE on LTP is a crucial endeavor to undertake, given that LTP is widely recognized as the fundamental mechanism underlying the establishment of learning and memory. Numerous studies have demonstrated that LTP in the hippocampus is impaired after SAE modeling, such as CLP, PCI, and LPS injection [49, 50, 52-54]. The observed phenomenon may potentially be attributed to alterations in glutamate receptors. Selective reductions in protein and mRNA levels of AMPAR and NMDAR subunits were observed in the hippocampus of both CLP and LPS injection sepsis models [53, 55-57]. Additionally, electrophysiological data from a study indicate that the combination of hypoxia and LPS stimuli synergistically triggers LTD in the rat hippocampal CA1 region [47]. Given the hypoperfusion of microcirculation observed in the pathophysiology of SAE, where local brain tissue is subjected to oxygen deprivation, it is plausible that LTD may also be involved [58].

Unfortunately, the clinical research documenting synaptic density and electrophysiological changes in patients with SAE is currently insufficient [59]. However, cerebrospinal fluid (CSF) data on synapse-associated proteins can reflect such changes [60, 61]. A recent study analyzed the expression of CSF proteins in patients with infected delirium and found that a panel of proteins related to synapse formation and function was downregulated compared to noninfectious patients [60]. Interestingly, alterations in the expression of neuron-microglia communication markers have been observed in CSF samples from patients with infectious delirium, indicating that dysregulation of microglial homeostasis may contribute to the pathophysiology of this condition [60]. Notably, researchers have recently investigated the impact of infant sepsis on neurological function by analyzing proteomic profiles in CSF samples obtained from clinically septic neonates [61]. The findings indicate a reduction in protein abundance, which is linked to the maturation of synaptic networks and plasticity. In the future, more specific biochemical indicators of CSF should be screened to evaluate cognitive function prognosis in patients with SAE.

## 4 POSSIBLE MECHANISMS OF SYNAPTIC CHANGES IN SAE

### 4.1 Glial Cells

The glial cells within the nervous system are essential for the healthy growth and operation of the brain. In the CNS, there exist three primary groups of glial cells: astrocytes, oligodendrocytes, and microglia [62]. Emerging evidence indicates that these cells have a vital function in the formation and maintenance of neural networks [63]. However, during inflammatory conditions, imbalances in cytokine and neurotrophic factor levels can disrupt synaptic plasticity and potentially harm neurons, with microglia playing a pivotal role in this process [20, 62]. This article will specifically focus on the contribution of glial cells, particularly microglia, to synaptic plasticity during the pathological progression of SAE.

#### 4.1.1 Participation in Synaptic Pruning

Synaptic pruning mediated by microglia is a fundamental mechanism for circuit refinement during brain development. Synaptic pruning refers to the process by which the brain eliminates excess synaptic connections to increase the efficiency of neuronal networks, and it occurs throughout the postnatal and adult stages of development [64]. The role of synaptic pruning has been validated in some neurodegenerative diseases [65], and recent evidence suggests its involvement in SAE as well.

Following peripheral LPS stimulation, the average volume of lysosomes in microglia increased [43], and the volume of internalized postsynaptic density protein (PSD95) significantly increased [66]. This suggests that the synaptic pruning by hyperactivated microglia is a crucial cause of the decrease in synaptic density during SAE [66]. The occurrence of this process may be related to the formation of spine-head filopodia (SHF) [66]. *In vivo* two-photon imaging of glial cell-synapse interactions showed that after LPS stimulation, the contact time between glial cells and neurons was prolonged. They approached dendritic spines and stretched and engulfed dendritic spine filopodia, forming SHF, which were significantly correlated with contact time and dendritic spine clearance rate [67]. This process may involve molecules, such as complement, phosphatidylserine (PS), and intercellular adhesion molecule-5 (ICAM-5), and is centered around the complement pathway.

The classic complement cascade has long been demonstrated to be essential in synaptic formation and pruning [68]. Under normal physiological conditions, microglia can regulate synaptic pruning through the classical complement cascade pathway. Complement proteins (primarily including C1q and C3) are specifically localized and bound to apoptotic, immature, or weakly developed synapses in the central nervous system. Furthermore, microglia express the complement receptor CR3, which identifies these proteins and triggers the phagocytosis of synapses. The cascade reaction is initiated by the C1q protein, while microglia or astrocytes serve as the primary sources of another complement protein, C3.

In the animal model of sepsis, the complement pathway is activated. After mice are attacked by bacteria or viruses, the upregulation of genes related to the complement pathway can be observed in the hippocampus [69]. The proteins produced are deposited around synapses [43], and the recruitment of SHF induced by microglia is intricately linked to the progression of this process [67]. The co-localization of C1 and the presynaptic terminal and C3 with microglia suggests that complement and microglia form an axis that allows microglia to phagocytose labeled synapses [66]. As the KO of C3 and C3 AR blocked the decrease in synaptic density, this indicates the necessity of the complement pathway for the function of microglia [69]. Reaffirmation of this conclusion was also observed through unbiased transcriptomics analysis conducted on hippocampal tissue and microglia isolated from septic mice [49]. It was found that genes related to lysosomal function (Ctse, Ctsl, and Lyz2), phagocytosis (Fcer1g, Lgals3, and C3), and complement receptors were upregulated in parallel with neuronal and synaptic damage [49].

Another signal reported to promote synaptic pruning is PS. ROS generated by oxidative stress promotes PS exposure on dendrites undergoing apoptosis or injury [70]. The triggering receptor expressed on myeloid cells 2 (TREM2) is a cell surface receptor on microglia that recognizes exposed PS [70], leading to the engulfment of tagged synapses [71]. The occurrence of neuroinflammation leads to oxidative stress in the brain parenchyma and an increase in ROS levels [72], so it is reasonable to speculate that PS-TREM2 is also involved in the synaptic pruning process in SAE. In addition, C1q can specifically recognize and bind to PS [73], and it is assumed that microglia engulfing dendrites marked by C1q may be related to PS-TREM2. However, more experiments are needed to verify this.

Currently, there are many controversies surrounding synaptic pruning in SAE. The first is whether microglia participate in synaptic plasticity regulation in SAE through synaptic pruning. Data from several studies have shown that after peripheral LPS stimulation, the CA3 synaptic density decreases, while the punctate density of PSD95 in microglial lysosomes has no significant changes in CA3 [43]. The second controversy is about the selectivity of microglia in engulfing synapses. Recent evidence suggests that after systemic LPS stimulation, the excitatory synaptic puncta in the hippocampal CA3 region covered by C3 are locally reduced, while the density of inhibitory synaptic puncta remains normal [43].

In contrast to this conclusion, Li *et al.* reported that LPS stimulation has no effect on the levels of excitatory synaptic associated proteins, such as synaptophysin and PSD95, but significantly reduces inhibitory synaptic associated proteins, including VGAT, GAD65/67, and gephyrin [74]. Blocking C3aR can rescue the loss of inhibitory synaptic-associated proteins [74]. The truth is still waiting to be gradually revealed. Given the potential temporal variability of synaptic effects induced by systemic inflammation, *in vivo,* imaging employing multi-photon microscopes can facilitate the direct observation of phagocytic actions performed by microglia on synapses during the process of synaptic pruning [75].

#### 4.1.2 Secretion of Soluble Molecules

Microglia can impact the nervous system not only through contact-dependent mechanisms, but also *via* the secretion of various factors with nutritional, transmitter, protective, or harmful effects. These factors include brain-derived neurotrophic factors, neurotransmitters (ATP and glutamate), several types of cytokines (*e.g*., IL-1β, IL-10, transforming growth factor-β(TGF-β)) [76], and microRNAs that directly affect gene expression [77]. In both physiological and pathological contexts, these substances mediate the interaction between microglia and synapses and participate in the regulation of synaptic plasticity. Microglia-derived soluble substances encompass direct secretion as well as extracellular vesicle formation, with microvesicles derived from microglia serving as vehicles for neurotoxic agents, such as IL-1β [78] and microRNAs [77].

Following peripheral inflammation stimulation, microglia can release a significant amount of inflammatory factors, including IL-1β, which has been extensively studied. While IL-1β plays a physiological role in cell defense, tissue repair, and neural regulation, its secretion during neuroinflammation has detrimental effects on synaptic plasticity. Both *in vitro* and *in vivo* studies have confirmed that microglial secretion of IL-1β affects synapses [79]. The expression of IL-1β in microglia of septic rats showed a significant up-regulation, concomitant with the presence of swollen and clumping synaptic vesicles near the presynaptic membrane in the cortex [80]. The level of SYN was decreased in primary cultured neurons incubated with IL-1β [80]. The research findings of Sheppard *et al.* provide additional substantiation for this perspective. LPS treatment can lead to a reduction in SYN and SYT in mouse hippocampal slices, but this effect can be prevented by depleting microglia prior to LPS exposure or pretreating with an IL-1β neutralizing antibody [81]. The p38-MAPK signaling pathway may mediate this process [79]. Recently, a study has confirmed that the activation of NLRP3 inflammasome in microglia is a mechanism for the secretion of IL-1β [78]. The recruitment of NLRP3 inflammasome and the activation of caspase-1 were induced by LPS-stimulated microglia [78]. Caspase-1 cleaves proinflammatory cytokine IL-1β, resulting in the release of its mature form, including IL-1β monomers and microvesicles rich in IL-1β [78].

During the upregulation of IL-1β expression, there is a concomitant downregulation in the expression of IL-10 and TGF-β in microglia. In the absence of external stimuli, microglial release of IL-10 can induce synapse formation, which is attenuated by neutralizing antibodies to IL-10 receptor. Following LPS treatment, this effect is further weakened [82]. This may be associated with the downregulation of IL-10 expression, and IL-1β is also implicated in this process, forming a complex regulatory network. This perspective is further supported in septic mice where intraperitoneal injection of LPS resulted in lower efficiency of IL-10-deficient mice compared to wild-type mice in hippocampus-dependent learning and memory tests [83]. However, it is worth noting that this mechanism may be more applicable to neonatal sepsis, as mature neurons do not express IL-10 receptors [83].

In a study investigating the composition of conditioned media from microglia and astrocytes under LPS stimulation, a reduction in TGF-β secretion was observed. Specifically, the level of TGF-β was significantly decreased in microglia but remained unchanged in astrocytes [48]. These findings were consistent with a decrease in both expression and release of TGF-β by activated microglia, while secretion of TGF-β by astrocytes appeared unaffected by these factors [84]. The diminished release of TGF-β, a molecule involved in the formation of synapses and produced by glial cells in the cerebral cortex, could potentially contribute to synaptic loss as a result of microglia activation.

Recent findings indicate that the interaction between neurons and microglia, as well as other glial cells, can occur *via* the release of extracellular vesicles (EVs). The data obtained from cell experiments confirmed that following stimulation by inflammatory factors (IL-1β, TNF-α, IFN-γ), microglia secrete EVs that transfer bioactive miRNAs to neurons *via* membrane fusion, thereby regulating the expression of synaptic-related genes (syt1, nlg1) and ultimately reducing dendritic spine density and excitatory synapses [77]. This phenomenon has also been confirmed *in vivo*. Releasing inflammatory EVs obtained from microglia into the hippocampus of mature mice has the potential to reduce the occurrence and intensity of mEPSCs in CA1 pyramidal cells, as well as reduce spine density. These findings demonstrate that glial cell-activated EVs may facilitate synaptic elimination, and it is plausible that microglia-derived EVs could impede synapse formation [77]. This process appears to be modulated by cytokines, such as IL-1β, TNF-α, and IFN-γ, which promote the release of microglial EVs. Since these cytokines have been shown to be upregulated in SAE brain tissue, it suggests a potential involvement of this mechanism in the development of SAE, necessitating further investigation.

### 4.2 Cytokine

In the healthy brain, physiologically low levels of inflammatory cytokines are required for remodeling neural circuits, fostering hippocampal LTP and promoting neurogenesis [85]. During SAE, the brain is in a state of neuroinflammation, and glial cells and other immune cells can secrete high levels of pro-inflammatory cytokines. The data obtained from clinical patients revealed elevated levels of multiple cytokines (such as IL-1, TNF-a, IL-8 and HMGB1) in the CSF following sepsis [86-88]. However, these findings exist variability among patients, and actual cytokine levels may differ due to various factors, including individual variances in the type and severity of infection. Besides, data from rodent studies have shown that LPS causes an increase in the production of inflammatory cytokines in the brain tissue [89]. The production of these inflammatory mediators disrupts the homeostasis required for neurophysiological activity and has an impact on synaptic plasticity. To provide a more comprehensive depiction, we have summarized the synaptic characteristics of the relevant cytokines in Table **2** under both physiological and SAE pathological conditions.

#### 4.2.1 IL-1β

The impact of IL-1β on synaptic plasticity relies on the dosage [90]. The brain typically exhibits low levels of IL-1 under physiological conditions. Avital *et al.* demonstrated that the presence of IL-1β at physiological levels is essential for both the initiation and maintenance of LTP, as supported by their observation that CA1 brain sections from mice lacking IL-1R exhibited a deficiency in LTP [91]. When IL-1β levels rise, an adverse effect on synaptic plasticity occurs. Elevated IL-1β levels have been observed in clinical SAE patients as well as in animal models of SAE. In the absence of clinical or microbiological signs of meningitis, IL-1β is elevated in plasma and CSF and has a high predictive accuracy for adverse clinical outcomes. In the hippocampus of septic mice, several data reports suggest that significantly higher levels of IL-1β 24 hours after sepsis. These levels persisted for 9 days and then decreased to control levels by 30 days. These findings are consistent with the timing of synapse loss in hippocampus of septic mice [92]. Under pathological settings, the function of IL-1β in modulating synaptic plasticity has drawn a lot of attention. A number of papers provided data demonstrating that IL-1β can affect the functional state, morphology, and number of synapses.

Y. Imamura first identified the role of IL-1β in the deficiency of LTP after SAE. After the CLP model was established, the expression of IL-1β and its receptor in the hippocampus was up-regulated, and the loss of LTP in the hippocampus was observed, which could be abolished by IL-1R1 antagonist [93]. Other studies have shown that the effect of IL-1β on LTP is mediated by IL-1R, which may be related to the p38-MAPK pathway [94]. In mechanically isolated hippocampal CA1 pyramidal neurons, the activation of p38-MAPK was observed in response to IL-1β [94]. Additionally, the effect of IL-1β on NMDA-induced outward currents (INMDA-OUT) could be significantly inhibited by p38 inhibitor [94]. Intracellular infusion of the active recombinant protein p38α resulted in a notable decrease in the average amplitude of INMDA-OUT [94].

Glia-mediated synaptic loss has also been associated with IL-1β. Normal cultured microglia increased the number of synapses between neurons in the cerebral cortex, while the number of synapses decreased by 50% after LPS stimulation. The level of IL-1β in the culture medium was increased, and the application of IL-1Ra completely prevented LPS-induced synaptic loss [92]. This suggests that IL-1β secreted by activated microglia can mediate synaptic loss in SAE. This requires the simultaneous activation of multiple pathways that require both presynaptic and postsynaptic activity [95], in which the p38-MAPK pathway may play an important role since IL-1β inhibited synaptophysin expression in primary neurons *via* the p38-MAPK signaling pathway [96].

A functional connection was also established between IL-1β and GABA-A receptors [97, 98]. The expression and phosphorylation of GABA-A receptors on the membrane surface were enhanced by IL-1β [97]. Consistent with these findings, IL-1β potentiates tonic inhibitory currents produced by GABA-A receptors expressing the α5 subunit in mouse hippocampal slices [98]. Changes in GABA-A receptors, along with concurrent modifications in neuronal currents, indicate that IL-1β enhances the GABAergic tone and modifies the efficacy of GABAergic synapses. This mechanism is thought to play a role in the development of memory impairments and encephalopathy in sepsis or other CNS disorders characterized by increased levels of IL-1β [99].

#### 4.2.2 HMGB1

The predominant subcellular localization of high mobility group box 1 protein(HMGB1) is within the nucleus under physiological conditions, where it exerts a crucial role in maintaining cellular homeostasis by regulating gene expression [100]. In the context of sepsis pathology, HMGB1 is a delayed mediator of sepsis-induced inflammation [101] and plays a role in amplifying neuroinflammation [102]. As a nuclear protein released by macrophages, its active release involves nucleoplasmic transfer as a crucial step [103, 104]. Data from both SAE patients and animal models have shown that the plasma level of HMGB1 increases after the peak release of early inflammatory factors [105-107]. After CLP, the decrease in nuclear HMGB1 levels also confirmed the increased release of HMGB1 [108]. The administration of anti-HMGB1 antibodies following SAE can enhance dendritic spine density and significantly ameliorate brain pathology and memory impairment [109]. This phenomenon suggests that HMGB1 plays a role in regulating synaptic plasticity. Different from the direct regulation mechanism of IL-1β through IL-1R, HMGB1 is involved in enhancing the phagocytic ability of microglia and inducing excitatory synaptic phagocytosis [108]. Interfering with HMGB1 release can reduce the synaptic elimination mediated by microglia [108].

The damage-associated molecular pattern HMGB1 exerts its effects through receptor binding, with the receptor for advanced glycation end products (RAGE) being one of these receptors. The expression of RAGE is upregulated in the hippocampus and prefrontal cortex during cognitive impairment 30 days after CLP [110]. Furthermore, the presence of disseminated intravascular coagulation and sepsis severity in patients is associated with increased serum levels of sRAGE [111]. HMGB1 interacts with RAGE, resulting in the activation of RAGE and subsequent induction of neuroinflammation through modulation of NF-kB signaling pathway and upregulation of early growth response protein-1 expression [112, 113]. Inhibition of HMGB1-RAGE signaling has the potential to alleviate cognitive impairment following SAE [114, 115]. These findings collectively suggest that the HMGB1-RAGE signaling pathway plays a role in regulating synaptic plasticity.

#### 4.2.3 TNF-a

Previous studies have reported that under physiological conditions, TNF-α plays a crucial role in the regulation of synaptic scaling. Synaptic scaling is a negative feedback regulatory mechanism of synaptic transmission, whereby neurons can increase their synaptic strength to maintain overall activity at a stable level when presynaptic terminal-driven synaptic transmission is reduced [116]. This process predominantly relies on AMPARs. When synaptic strength decreases, astrocyte-released TNF-α activates TNFR1, resulting in transient insertion of AMPARs into the postsynaptic membrane to enhance synaptic transmission [117].

It is worth noting that the precise mechanism underlying the impact of TNF-a on synaptic plasticity in SAE animal models remains unclear, with only one study suggesting its potential involvement in this process. The findings from this investigation revealed that CLP mice exhibited impaired memory for recognizing novel objects after 10 days of modeling. However, such deficits were not observed in TNFR1 knockout mice [118]. One plausible hypothesis is that exposure to elevated levels of TNF-a in SAE brain tissue may result in an upregulation of AMPAR on the cell surface, thereby leading to excitotoxic damage and a reduction in spinous processes. Undoubtedly, further research is warranted to delve into this matter.

### 4.3 Regulation of Microglia and Cytokine to Reduce Synaptic Plasticity Dysfunction in SAE

The brain employs multiple pathways to regulate the role of neuroinflammation in synaptic plasticity, thereby exerting neuroprotective effects. ICAM-5 is a crucial molecule that plays a protective role by being released from neurons as a soluble protein in an activity-dependent manner and binding to microglia [119]. *In vitro* studies have shown that ICAM-5 promotes the downregulation of adhesion and phagocytosis of microglia, thus reducing the occurrence of synaptic pruning [120]. In addition, it can decrease the secretion of pro-inflammatory cytokines TNF-α and IL-1β while inducing the secretion of anti-inflammatory cytokine IL-10 in microglia stimulated by LPS, thereby promoting their transformation into an anti-inflammatory phenotype [120]. The regulatory impact of ICAM-5 is contingent upon the expression of the integrin β2 chain by microglia [120]. Releasing ICAM-5 could potentially serve as a mechanism for highly active synapses to prevent themselves [121].

As a classic neurotransmitter, norepinephrine (NE) is also involved in the regulation of microglia. Gyoneva and Trayneils first demonstrated the effect of NE on the morphological and process motility of microglial cells [122]. In 2019, *in vivo* imaging of acute cortical slices revealed that β2 adrenergic receptor (β2-AR) mediates NE signaling to modulate microglial process dynamics [123]. In septic mice, both early and delayed administration of β2-AR agonists can ameliorate sepsis-induced cognitive impairment, potentially through the inhibition of microglial process extension and synaptic pruning *via* NE stimulation [124]. Furthermore, early treatment with β2-AR agonists may promote the differentiation of microglia into anti-inflammatory phenotypes, reduce pro-inflammatory cytokine secretion (including IL-1β and TNF-α), and mitigate synaptic damage [124].

The intervention of microglia and cytokines presents a promising therapeutic approach for SAE. Substantial evidence from animal experiments supports the efficacy of pharmacological intervention in mitigating impaired synaptic plasticity associated with SAE, thereby enhancing cognitive function. As previously stated, caspase-1 plays a crucial role in the secretion of IL-1β. In the mouse model of sepsis, inhibition of caspase-1 can provide neuroprotection by reducing microglial activation and attenuating serum and brain expression of IL-1β and TNF-α [125]. It mitigated the decrease in synapse-related protein expression and loss of dendritic spines after SAE, preserved LTP, and improved cognitive function in mice [125].

Minocycline (MINO), a broad-spectrum tetracycline antibiotic, has garnered research attention not only for its antibacterial properties but also for its anti-inflammatory effects in the treatment of SAE [126]. Evidence indicates that MINO can inhibit microglial activation, reduce the expression of pro-inflammatory cytokines (including IL-1β, TNF-α, and IL-6) in the brain [127-130], and ameliorate cognitive dysfunction in CLP animal models [127]. Further investigations demonstrated that MINO has the potential to mitigate sepsis-induced LTP impairment in the hippocampus of mice [128]. This effect may be attributed to its ability to suppress IL-1R-mediated signaling, as the protective impact of IL-1R antagonists on LTP was abrogated following MINO administration [128]. It should be noted that in the majority of experimental models, the dosages of MINO utilized for neuroprotection exceed those employed for infection treatment [128-130]. The determination of a safe human dosage capable of achieving cognitive improvement remains to be established, and its clinical application will necessitate extensive exploration over an extended period. Similar to MINO, galantamine [131], tauroursodeoxycholic acid [132], and Trichosanthis Semen and Zingiberis Rhizoma Mixture [133] also reduced hippocampal synaptic protein loss and increased dendritic spine density through their anti-inflammatory effects.

Interestingly, the post-SAE recovery environment also exerts an impact on synaptic plasticity and cognitive function. In experimental settings, an enriched environment (EE) refers to a larger cage area with more animals and facilities, such as toys and tunnels, allowing for complex social interactions among animals [134]. The efficacy of EE in reducing neuroinflammation and mitigating synaptic plasticity damage has been demonstrated in CLP and LPS injection models [135-137]. These beneficial effects are mediated through the binding of vasopressin (VP) to VP receptor 1a [137]. In essence, interventions aimed at improving environmental conditions may yield more favorable long-term neurobehavioral outcomes. However, the utilization of EE as a rehabilitation method for cognitive rehabilitation in patients with SAE is infrequently observed in clinical practice. Hence, there is an urgent requirement for a large-scale, randomized, controlled prospective study. Based on the current research demonstrating the role of VP in improving EE-induced SAE-related learning and memory deficits, VP could be regarded as a promising therapeutic agent for managing the consequences of SAE. Augmenting endogenous VP synthesis or supplementing exogenous VP may serve as a viable alternative approach. To gain a deeper understanding of the impact of neuroinflammation on synaptic plasticity, we have provided a summary of Fig. (**1**).

## 5 ANIMAL MODELS OF SAE

According to the mechanism discussed above, we summarized the animal models currently used. Sepsis induction in animal models primarily involves the administration of toxic reagents (*e.g*., LPS), live pathogen injection (bacteria, fecal fluid, *etc*.), and disruption of barrier integrity (*e.g*., CLP) [138, 139]. In studies on synaptic plasticity in SAE, CLP and LPS injections are commonly utilized due to their respective advantages.

The CLP model has long been regarded as the gold standard for sepsis research due to its similarity to the pathophysiological process of human sepsis [140]. However, the method is difficult to standardize due to variations in surgical parameters, such as cecal ligation length, needle size, and puncture times [138, 140, 141]. Additionally, CLP-induced septic mice exhibit severe motor dysfunction, and the evaluation of early cognitive impairment is restricted, rendering it unsuitable for investigating acute SAE-related cognitive dysfunction. The injection of LPS is a commonly employed method due to its simplicity and reproducibility [141]. The type of inflammation induced varies depending on the administration route utilized. Intraperitoneal injection induces systemic inflammation, while lateral ventricular injection focuses on neuroinflammation induction. Additionally, examining electrophysiology after perfusion with LPS in animal brain slices provides an alternative research approach [47]. Further, it is important to note that the variations in molecular composition of different serotypes of LPS result in varying levels of neuroinflammation and diverse effects on synapses within the brain [43, 52].

## CONCLUSION

The long-term cognitive dysfunction and the changes in the mental state of SAE patients are major challenges in the current treatment process, and there are no effective prevention and treatment methods. Given the crucial role that synaptic plasticity plays in cognitive processes, this review focuses on elucidating the alterations in synaptic plasticity associated with SAE while also providing a comprehensive overview of the roles played by glial cells and cytokines in this intricate process. Due to the pivotal role of glial cells in this process, intervening in the interaction between glial cells and synapses could potentially offer a novel therapeutic avenue. Besides, the existing means of directly targeting synapses and their potential impact on the prognosis of SAE have not been reported. Therefore, it is crucial to note that further research is required due to the potential disruption of normal neural function associated with synaptic targeting. We are optimistic that further research on synapses will provide innovative perspectives for the treatment of SAE.

## Figures and Tables

**Fig. (1):**
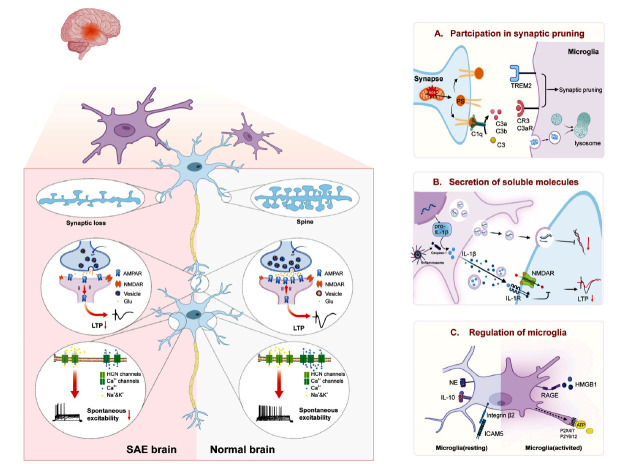
The Role of Neuroinflammation in Synaptic Plasticity of SAE. During SAE, neuroinflammation leads to a reduction in synaptic density and impaired LTP, while LTD is enhanced. Microglia can modulate synaptic plasticity through both contact-dependent pruning and non-contact secretion of soluble molecules. (**A**) Microglia participate in synaptic pruning. Mitochondrial oxidative stress induces the generation of ROS, which in turn promotes the externalization of PS. The externalized PS then binds to C1q, leading to the cleavage of C3 and subsequent synaptic pruning *via* CR3 and C3AR. PS-TREM2 is also involved in this process. (**B**) Microglia secrete soluble molecules. Microglia have the ability to secrete both IL-1β monomers and IL-1β-containing vesicles, which can impact the postsynaptic membrane potential in an IL-1R-dependent manner. Meanwhile, miRNA-containing vesicles can be secreted to modulate the gene expression of synapse-associated proteins. (**C**) Multiple pathways can regulate microglia. ICAM-5, IL-10 and NE inhibit the phagocytosis of microglia and the expression of pro-inflammatory cytokines. The opposite was observed for HMGB-1. **Abbreviations**: SAE: sepsis-associated encephalopathy; LTP: long-time potentiation; LTD: long-term depression; ROS: reactive oxygen species; PS: phosphatidylserine; TREM2: triggering receptor expressed on myeloid cells 2; NMDA: N-Methyl-D-aspartic acid; NE: norepinephrine; ICAM-5: intercellular adhesion molecule-5; HMGB-1: High mobility group box 1 protein; RAGE: the receptor for advanced glycation end products (Created with BioRender.com).

**Table 1:** Synaptic changes in animal models of SAE.

**Year**	**Animal**	**Treatment**	**Synapse Changes**	**References**
2023	C57BL/6J mice (8-9 weeks)	CLP	Both neuronal activity and spine density were significantly reduced in hippocampal CA1 of CLP mice.	[50]
2023	C57BL/6J mice (10-13 weeks)	PCI (3.5 μl/g, i.p.)	The frequency of mEPSCs in hippocampal CA1 pyramidal neurons was reduced. SYT and PSD95 levels decreased after 3 days of modeling, but returned to baseline after 10 days; mice exhibited impaired memory function.	[49]
2023	C57BL/6JGpt mice (10-12 weeks)	CLP	The morphology of pyramidal neurons in the CA1 region of the hippocampus exhibited impairments, with a significant reduction in dendritic spine density and compromised synaptic structure integrity.	[108]
2021	C57BL/6N mice (14-16 months)	LPS (1.5 mg/kg, i.p.)	After 63 days of injection, excitatory synaptic sites in the CA3 region of the hippocampus were locally reduced, and the density of inhibitory synaptic sites remained normal.	[43]
2021	SD Rats (8 weeks)	CLP	After high-frequency stimulation, a significant decrease in fEPSP slope was observed, accompanied by the down-regulation of presynaptic proteins SYN and SYT as well as postsynaptic proteins NR2B, GluR1, and PSD95.	[53]
2021	Swiss-Webster mice (Pregnant)	*Klebsiella pneumoniae* (3X108 CFU, 50 μl)	Decreased levels of SYT and PSD95 in the hippocampus, neocortex, frontal cortex, and cerebellum, and pups showed motor impairment, depression-like behavior, fear-conditioned memory, and learning deficits.	[142]
2020	C57BL/6J mice (12 - 14 weeks)	LPS (2 mg/kg, i.p.)	LPS selectively induces the loss of inhibitory but not excitatory synapse-associated proteins of the hippocampus.	[74]
2020	C57BL/6 mice (4 and 13 months)	LPS (0.2 mg/kg, i.p.)	At 3 months after injection, hippocampal dendritic spine density was normal in young mice and decreased slowly in old mice. Deficits in LTP can be observed.	[52]
2019	Kunming mice (7-8 weeks)	LPS (10 mg/kg, i.p.)	The expression of SYT showed a significant decrease, while there was no notable difference observed in the level of PSD95 expression.	[66]
2019	C57BL/6 mice (3-4 months)	CLP	The reduction in the expression of PSD95 and GluN2B is exhibited, which was accompanied by hippocampus-dependent cognitive deficits.	[124]
2019	SD rats	LPS (1 mg/kg, i.p.)	The expression of SYT and NMDAR was significantly reduced in the hippocampus of the septic brain. Consistent with this, the number of dendritic spines associated with pyramidal neurons was decreased in the hippocampal CA1 region 28 days after LPS administration. The reduction in both synaptic vesicles and synapses, as well as swelling, were observed.	[143]
2019	C57BL/6 mice (3-4 months)	LPS (3 mg/kg, i.p.)	The levels of PSD95 were significantly reduced in the hippocampus, prefrontal cortex, and striatum 24 hours after LPS injection, with the longest duration observed in the hippocampus. Conversely, there was no change in synaptophysin levels.	[57]
2017	SD rats (1 day)	LPS (1 mg/kg, i.p.)	The expression of SYT was reduced, while the protein expression of PSD95 remained relatively unchanged. Electron microscopy revealed altered synaptic morphology, characterized by swelling and aggregation of synaptic vesicles near the presynaptic membrane.	[79]
2017	C57BL/6 mice (2 months)	CLP LPS (8 mg/kg, i.p.)	Impaired spatial memory and transient reduction in NMDA and AMPA receptor expression in the hippocampus were observed without concomitant neuronal cell death.	[144]
2015	Swiss mice (8-12 weeks)	CLP	Nine days post-modeling, mice displayed memory impairments and reduced numbers of excitatory synapses in the hippocampus and cortex, as evidenced by co-localization of SYT/PSD95. Both behavioral deficits and SYT/ PSD95 co-localization returned to baseline levels within 30 days following sepsis.	[48]
2015	Wistar rats	LPS (6 mg/kg, i.p.)	LTP is inhibited in CA1 pyramidal neurons of the hippocampus.	[54]
2014	SD rats (18-23 days)	LPS (10 ug/ml, Hippocampal brain slice perfusion)	Perfusion of LPS did not alter basal synaptic transmission. Hypoxia caused immediate transient inhibition of fEPSP, which was completely reversed after normoxic reperfusion.	[47]
2012	BALB/c mice (6-8 weeks)	CLP	Sustained reduction in dendritic spine density in CA1 region not accompanied by neuronal cell death, long-term spatial memory as well as learning deficits in mice.	[109]
2011	C57BL/6 mice (2-3 months)	LPS (0.5 mg/kg, i.p.)	The density of dendritic spines in the somatosensory cortex showed a significant decrease 8 weeks after LPS treatment.	[45]
2009	C57BL/6 mice (3 months)	LPS (5 mg/kg, i.p.)	Increased working memory errors and chronic reduction of synaptic proteins in the hippocampus 2 months after injection, but no neuronal cell death.	[145]

**Abbreviations:** PCI: peritoneal contamination and infection; PSD95: postsynaptic density protein 95; CLP: cecal ligation and puncture; LPS: lipopolysaccharide; SYN: Synapsin; SYT: Synaptophysin; LTP: long-time potentiation; NMDA: N-Methyl-D-aspartic acid; AMPA: alpha-amino-3-hydroxy-5-methyl-4-isoxazole-propionic acid.

**Table 2:** The synaptic properties of the main cytokines implicated in SAE pathology.

**Cytokines**	**Physiological Condition**	**Pathophysiological Condition of SAE**
IL-1β	Regulation of basal synaptic transmission: The binding of IL-1β to IL-1R1 promotes phosphorylation of NR2B, enhances the flow of Ca^2+^ through NMDAR, and increases neuronal excitability [146]. Involvement in LTP formation: hippocampal LTP is impaired after inhibition of IL-1R [147]. Maintenance of the dendritic spine stability [148].	Involvement in hippocampal LTP impairment: CLP induced a reduction in hippocampal LTP, which was restored upon administration of IL-1R antagonists [149]. Mediating microglia-induced synaptic loss: IL-1R antagonists mitigate synaptic loss of microglial following LPS stimulation [150]. Enhancement of the GABAergic tone and the efficacy of GABAergic synapses [151].
HMGB1	No related articles have been seen.	Mediating microglia-induced synaptic loss: The inhibition of HMGB1 secretion resulted in a decrease in the phagocytic capacity of microglia and a reduction in synaptic loss [152].
TNF-a	Involvement in synaptic scaling: The reduction of synaptic strength leads to the binding of TNF-a, secreted by astrocytes, to TNFR1 and subsequently enhances the expression of AMPAR on the synaptic membrane [117].	The TNFR1-knocked-out mice exhibited no memory impairment after 10 days of CLP, in contrast to the control group [118].

## LIST OF ABBREVIATIONS

AMPAR: Alpha-amino-3-hydroxy-5-methyl-4-isoxazole-propionic Acid Receptor
BBB: Blood-brain Barrier
β2-AR: β2 Adrenergic Receptor
CLP: Cecal Ligation and Puncture
CNS: Central Nervous System
CSF: Cerebrospinal Fluid
EE: Enriched Environment
EVs: Extracellular Vesicles
HMGB1: High Mobility Group Box 1 Protein
ICAM-5: Intercellular Adhesion Molecule-5
IL-1: Interleukin-1
IL-6: Interleukin-6
LPS: Lipopolysaccharide
LTD: Long-term Depression
LTP: Long-term Potentiation
MINO: Minocycline
NE: Norepinephrine
NMDAR: N-methyl-d-aspartic Acid Receptor
PCI: Peritoneal Contamination And Infection
PS: Phosphatidylserine
PSD-95: Postsynaptic Density Protein 95
RAGE: Receptor for Advanced Glycation End Products
ROS: Reactive Oxygen Species
SAE: Sepsis-associated Encephalopathy
SHF: Spine-head Filopodia
SYN: Synapsin
SYT: Synaptophysin
TGF-β: Transforming Growth Factor-β
TNF-α: Tumor Necrosis Factor-α
TREM2: Triggering Receptor Expressed on Myeloid Cells 2
VP: Vasopressin
